# The role of light sheet microscopy for non-invasive imaging of live embryos

**DOI:** 10.1093/humrep/deaf091

**Published:** 2025-05-15

**Authors:** Kylie R Dunning, Kishan Dholakia

**Affiliations:** Centre of Light for Life, University of Adelaide, Adelaide, Australia; The Robinson Research Institute, School of Biomedicine, University of Adelaide, Adelaide, Australia; Centre of Light for Life, University of Adelaide, Adelaide, Australia; School of Biological Sciences, University of Adelaide, Adelaide, Australia; SUPA, School of Physics and Astronomy, University of St Andrews, North Haugh, Fife, UK

Dear Editors,

We write with regard to the paper by Vargas-Ordaz *et al.* (2025) recently published in *Human Reproduction*. The study describes light-sheet imaging of pre-implantation mouse embryos in an on-chip environment, capturing autofluorescence from NAD(P)H, at an excitation wavelength of 405 nm. This fluorescence is derived primarily from the reduced forms of nicotinamide adenine dinucleotide (NADH) and nicotinamide adenine dinucleotide phosphate (NADPH). Within embryos, NADH and NADPH play critical roles as co-factors in cellular metabolism and redox balance, driving energy production and biosynthetic processes essential for development. Thus, capturing autofluorescence from NAD(P)H alone as performed in Vargas-Ordaz *et al.* (2025), or in combination with other metabolic co-factors (Morizet *et al.*, 2023; Chow *et al.*, 2024a) may be informative of embryo viability.

The Vargas-Ordaz *et al.* paper forms part of a wider portfolio of new optical methods that have emerged and aim to non-invasively assess embryo developmental potential. The authors however make statements that either omit, or inaccurately portray, key literature in this field for the use of light sheet fluorescence microscopy (LSFM) for embryo imaging. This may inadvertently misrepresent this exciting technology to other proponents wishing to engage in this field. In particular, they state: ‘However, there has been no investigations using LSFM in embryos to achieve metabolic imaging by single-photon exciting endogenous cell fluorophores such as NAD(P)H as the LSFM adoption in mouse embryo development has been hindered by the challenge of suspending the sample between objectives, which often involves the use of a gel confinement’.

LSFM has rapidly risen to be the method of choice for a range of studies in developmental biology. This has been accelerated by the recognition that it is very fast and exhibits very low phototoxicity, including pertinently for the pre-implantation embryo (Chow *et al.*, 2024b; Vargas-Ordaz *et al.*, 2025). In fact, a study published in 2023, that predates this present paper, successfully utilized LSFM for metabolic imaging of mouse embryos (Morizet *et al.*, 2023). This was realized by capturing autofluorescence from the intracellular metabolic cofactors NAD(P)H and flavin adenine dinucleotide. Imaging of multiple metabolic co-factors was achieved using ‘single-photon’ excitation at a sole wavelength of 375 nm (Morizet *et al.*, 2023). Recording the abundance of these metabolic co-factors enabled calculation of the optical redox ratio—a non-invasive indicator of cell metabolism. Cell metabolism was found to be dynamic, significantly increasing throughout preimplantation development across the stages investigated: 2-cell, 4-cell, 8-cell, morula, and blastocyst (Morizet *et al.*, 2023). This study demonstrated successful non-invasive monitoring of metabolism in a spatial and temporal manner that may be indicative of embryo viability. These investigations confirm that ‘single photon’ LSFM using standard optics (free space) is a viable tool for non-invasive metabolic imaging of the embryo, and predates the Vargas-Ordaz *et al.* study (2025).

Though suspending samples for LSFM imaging can pose challenges, particularly in the context of gel confinement, researchers have successfully developed alternative mounting techniques and optimized imaging setups to mitigate these issues for live embryos (Strnad *et al.*, 2016; Reichmann *et al.*, 2018; Morizet *et al.*, 2023). Strnad *et al.* (cited by Vargas-Ordaz *et al.*, though not discussed in context) demonstrated this by employing high-resolution water immersion objectives in conjunction with a custom-designed chamber. This set-up enabled longitudinal imaging of mouse embryos within a standard microdrop of culture medium under oil, while ensuring that temperature and atmospheric conditions were maintained for optimal embryo development. The same team demonstrated that LSFM is able to image multiple embryos in parallel (Strnad *et al.*, 2016; Reichmann *et al.*, 2018), paving the way for high-throughput analysis of embryos in separate microdrops of media under oil.

Previous LSFM studies have therefore conducted both ‘single-photon’ metabolic imaging and indeed overcome any issues related to sample mounting for embryos. The study by Vargas-Ordaz *et al.*, does extend this topic area to a microfluidic environment. Whether the field will benefit from this step or might leverage the already established advances using ‘standard’ LSFM remains to be seen. It is an exciting and important juncture in the field.
